# Development and application of a transcriptional sensor for detection of heterologous acrylic acid production in *E. coli*

**DOI:** 10.1186/s12934-019-1185-y

**Published:** 2019-08-19

**Authors:** Sarada S. Raghavan, Sharon Chee, Juntao Li, Jeremie Poschmann, Niranjan Nagarajan, Siau Jia Wei, Chandra S. Verma, Farid J. Ghadessy

**Affiliations:** 1 p53 Laboratory Technology Development Group, A*STAR, 8A Biomedical Grove #06-06 Immunos, Singapore, 138648 Singapore; 2 Genome Institute of Singapore, 60 Biopolis Street, Genome, #02-01, Singapore, 138672 Singapore; 3 Centre de Recherche en Transplantation et Immunologie, Inserm, CHU-Nantes, Nantes, France; 4 Bioinformatics Institute, A*STAR, 30 Biopolis Street, Singapore, 138671 Singapore; 5 Department of Biological Sciences, National University of Singapore, 14 Science Drive 4, Singapore, 117543 Singapore; 6 School of Biological Sciences, Nanyang Technological University, 60 Nanyang Drive, Singapore, 637551 Singapore

## Abstract

**Background:**

Acrylic acid (AA) is a widely used commodity chemical derived from non-renewable fossil fuel sources. Alternative microbial-based production methodologies are being developed with the aim of providing “green” acrylic acid. These initiatives will benefit from component sensing tools that facilitate rapid and easy detection of in vivo AA production.

**Results:**

We developed a novel transcriptional sensor facilitating in vivo detection of acrylic acid (AA). RNAseq analysis of *Escherichia coli* exposed to sub-lethal doses of acrylic acid identified a selectively responsive promoter (P_yhcN_) that was cloned upstream of the eGFP gene. In the presence of AA, eGFP expression in *E. coli* cells harbouring the sensing construct was readily observable by fluorescence read-out. Low concentrations of AA (500 μM) could be detected whilst the closely related lactic and 3-hydroxy propionic acids failed to activate the sensor. We further used the developed AA-biosensor for in vivo FACS-based screening and identification of amidase mutants with improved catalytic properties for deamination of acrylamide to acrylic acid.

**Conclusions:**

The transcriptional AA sensor developed in this study will benefit strain, enzyme and pathway engineering initiatives targeting the efficient formation of bio-acrylic acid.

**Electronic supplementary material:**

The online version of this article (10.1186/s12934-019-1185-y) contains supplementary material, which is available to authorized users.

## Background

Acrylic acid (AA; also known as 2-propenoic acid) is a low molecular weight commodity chemical synthesized from petroleum-derived products. It is a feedstock for acrylate esters that are extensively used in the manufacture of paint-additives, adhesives, textiles and super absorbent materials such as diapers. Acrylic acid is conventionally generated by oxidation of propylene or propane, and the main challenge to this process is dependence on non-renewable fossil-fuel sources subject to unpredictable price fluctuations. Alternative “green” bio-based routes to produce acrylic acid from renewable sources such as sugars have therefore been proposed and investigated [1–4].

Early research focused on endogenous acrylic acid producers, such as the obligate anaerobes *Clostridium propionicum* and *Megasphaera elsdenii* that are capable of reducing lactic acid to propionic acid via an acrylyl-CoA intermediate [5]. In the presence of an electron acceptor (e.g. oxygen or methylene blue) acrylate can accumulate in *Clostridium propionicum* via oxidation of propionate [2, 6]. More recently, it was demonstrated that acrylate pathway enzymes from *Clostridium propionicum* can be expressed in a heterologous host (*E. coli),* enabling propionic acid biosynthesis from d-lactic acid via an acrylate intermediate [7]. Total biosynthesis of AA has further been described in engineered *E. coli* via enzymatic dehydration of 3-hydroxy propionic acid (3-HP) [8, 9]. However, given the low AA yields reported, significant pathway and enzyme engineering will be required to develop a commercially viable process. This endeavor will be expedited by tools facilitating rapid analysis of AA, routinely detected by low throughput, time consuming chromatographic techniques requiring sample preparation. High throughput screening campaigns interrogating sizeable mutant enzyme diversity and metabolic pathway iterations would particularly benefit from such tools. To this end, genetically encoded transcriptional sensors have proven useful for the clonal, real-time, and quantitative assessment of intracellular analyte concentrations [10]. These typically comprise cognate promoter elements of an analyte-responsive transcription factor that drive expression of a coupled read-out module, often a fluorescent protein [11]. Using this approach, sensors have been developed for a diverse array of analytes including amino acids, alcohols, flavonoids, organic acids, sugars and antibiotics [10–16]. When read-outs are compatible with high-throughput analytical platforms such as FACS, these sensors have facilitated significant improvements in product yields.

Here, we employ transcriptome analysis to identify *E. coli* genes selectively up-regulated in the presence of acrylic acid. Candidate promoters regulating these genes were coupled to an eGFP reporter module and sensitivity to acrylic acid confirmed. We further validate the lead sensor by in vivo selection of amidase variants showing improved catalytic conversion of acrylamide to acrylic acid.

## Results and discussion

### Identification of AA-responsive genes by transcriptome analysis

**Fig. 1:**
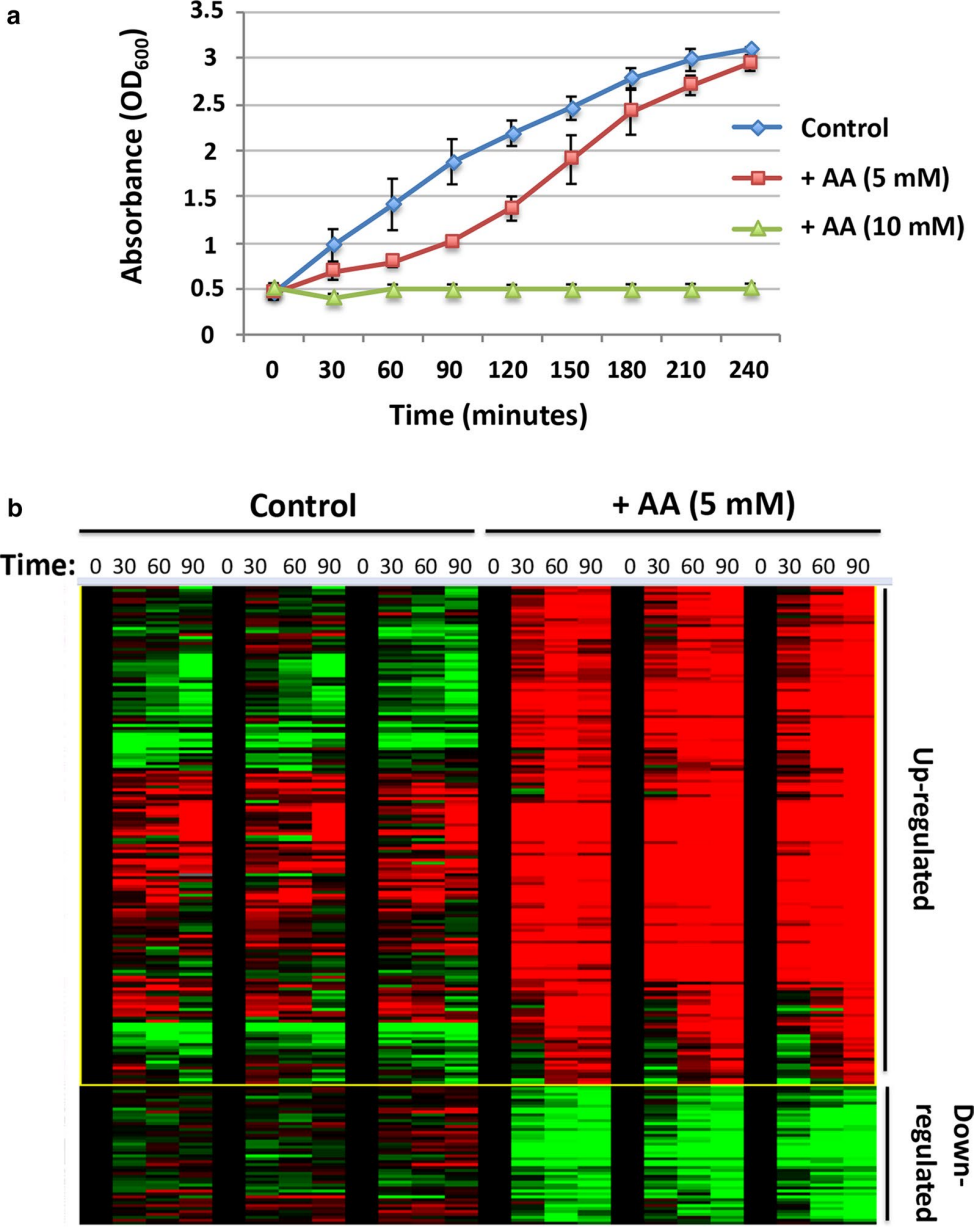
**a** Growth curves of *E. coli* cells treated with indicated concentrations of acrylic acid. Values represent average ± SD (n = 2). **b** RNAseq cluster heatmap indicating significant changes in up-regulated (red) and down-regulated (green) genes in *E. coli* exposed to acrylic acid (5 mM). Experiments were carried out in triplicate over the four indicated time points

*Escherichia coli* growth curves in the presence of acrylic acid indicated that concentrations > 5 mM were lethal. At 5 mM AA, cells recover after an initial delay (~ 90 min), and follow the growth characteristics of untreated cells (Fig. 1a). In order to study gene expression changes upon exposure to acrylic acid, we carried out RNAseq analysis of *E. coli* cells treated with 5 mM AA at early time points (0, 30, 60, 90 min) on the growth curve. Notable variations were observed in global expression profiles between treated and untreated samples (Fig. 1b). Greater than twofold transcriptional up-regulation occurred in 34, 58 and 83 genes at the respective 30, 60 and 90 min time points (Additional file 1).

### Validation of the acrylic acid—responsive genes

**Fig. 2:**
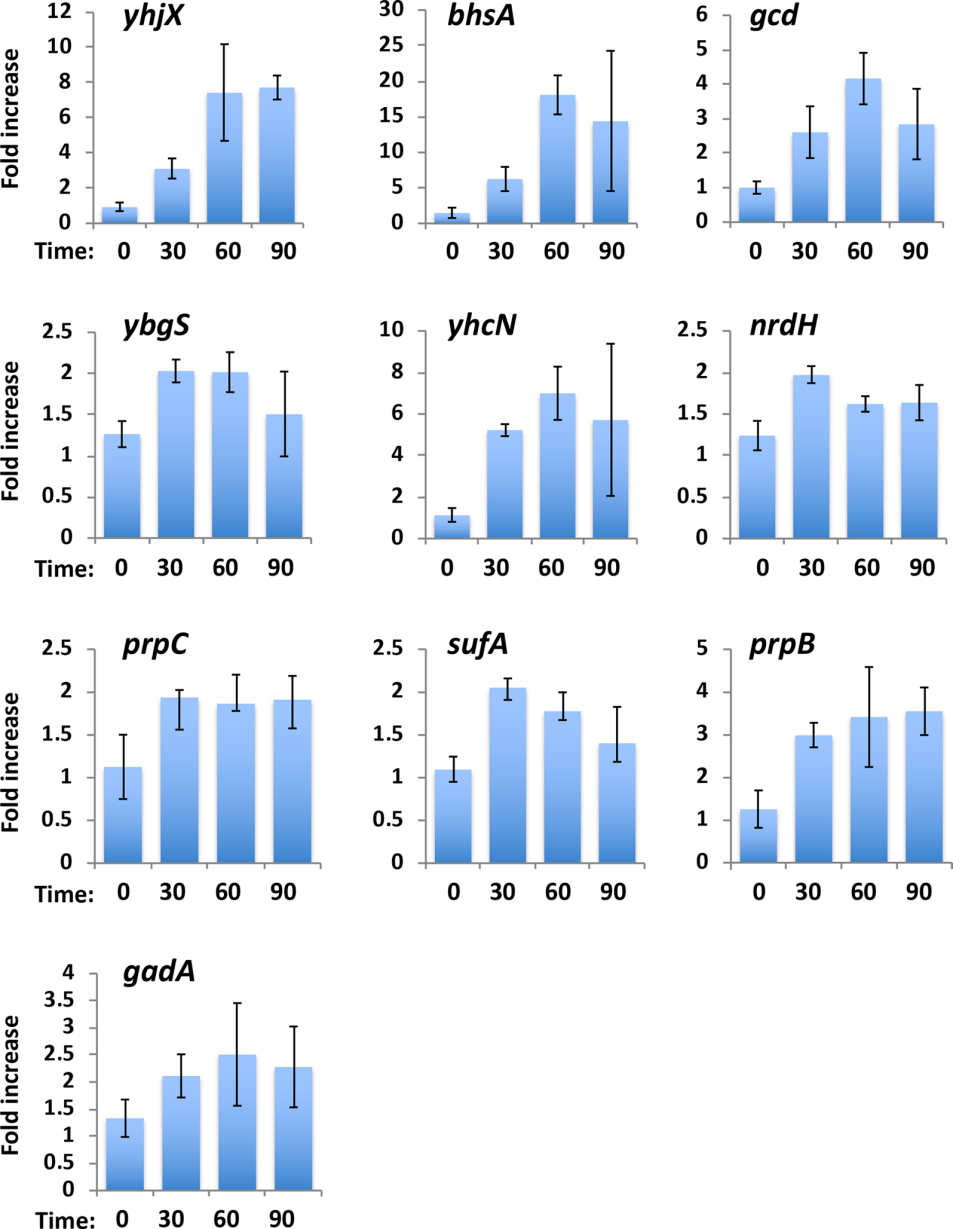
Transcription levels of candidate AA-responsive genes in *E. coli* measured by qPCR over indicated times (minutes) after AA treatment (5 mM). Values represent fold increase over levels of the *adk* housekeeping gene transcript (n = 3 ± SD)

Transcript levels of the top 10 genes up-regulated by AA at the 30 min sampling point (Table 1) were further investigated by quantitative real time PCR. In all cases, transcriptional up-regulation induced by AA was observed, with maximum increments over controls ranging between 2- and 18-fold (Fig. 2). Signals plateaued at 30–60 min for all genes, correlating with the 90 min time interval post AA-treatment when *E. coli* growth reverts to normal. A subset of these genes (*yhjX, bhsA, yhcN and prpB*) displayed improved signal to noise ratios due to persistent low level activity in the absence of AA (Additional file 2: Fig. S1), and were further evaluated.

**Table 1:** Top 10 genes upregulated after 30 min in *E. coli* exposed to acrylic acid

Gene	Fold up-regulation	Function
*yhjX*	6.5	Putative carboxylic acid antiporter
*bhsA*	5.7	Multiple stress protein
*gcd*	5.4	Glucose dehydrogenase
*ybgS*	5.4	Unknown
*yhcN*	4.5	Cellular response to acidity/peroxide
*nrdH*	4.3	Glutaredoxin-like protein
*prpC*	4.3	Citrate synthase
*sufA*	4.0	Iron-sulphur cluster assembly
*prpB*	4.0	Methyl isocitrate lyase
*gadA*	3.8	Glutamate decarboxylase

### Development of transcriptional sensor detecting acrylic acid

Promoter sequences of the 4 candidate genes (P_yhjX_, P_bhsA_, P_yhcN_ and P_prpB_) (Additional file 2: Fig. S2) were cloned upstream of eGFP and *E. coli* cells transformed with plasmids encoding the putative sensors. Cells were treated with 5 mM acrylic acid and fluorescence monitored over 24 h (Additional file 2: Fig. S3). The P_yhcN_, P_prpB_ and P_yhjX_ sensors displayed varying increases in fluorescence over no treatment and lactic acid controls. This was most pronounced for P_yhcN_, exhibiting ~ 50% increased signal over controls 2 h post-treatment that persisted over the 24 h time course. Relatively lower signal gains for the P_prpB_ and P_yhjX_ sensors (15–20%) were observed from the 6 h time point onwards. Efforts to improve the signal to noise ratio for the P_yhcN_-eGFP sensor by truncation of the yhcN promoter sequence did not boost sensor performance (Additional file 2: Fig. S4).

### Specificity and sensitivity of the acrylic acid sensor

**Fig. 3:**
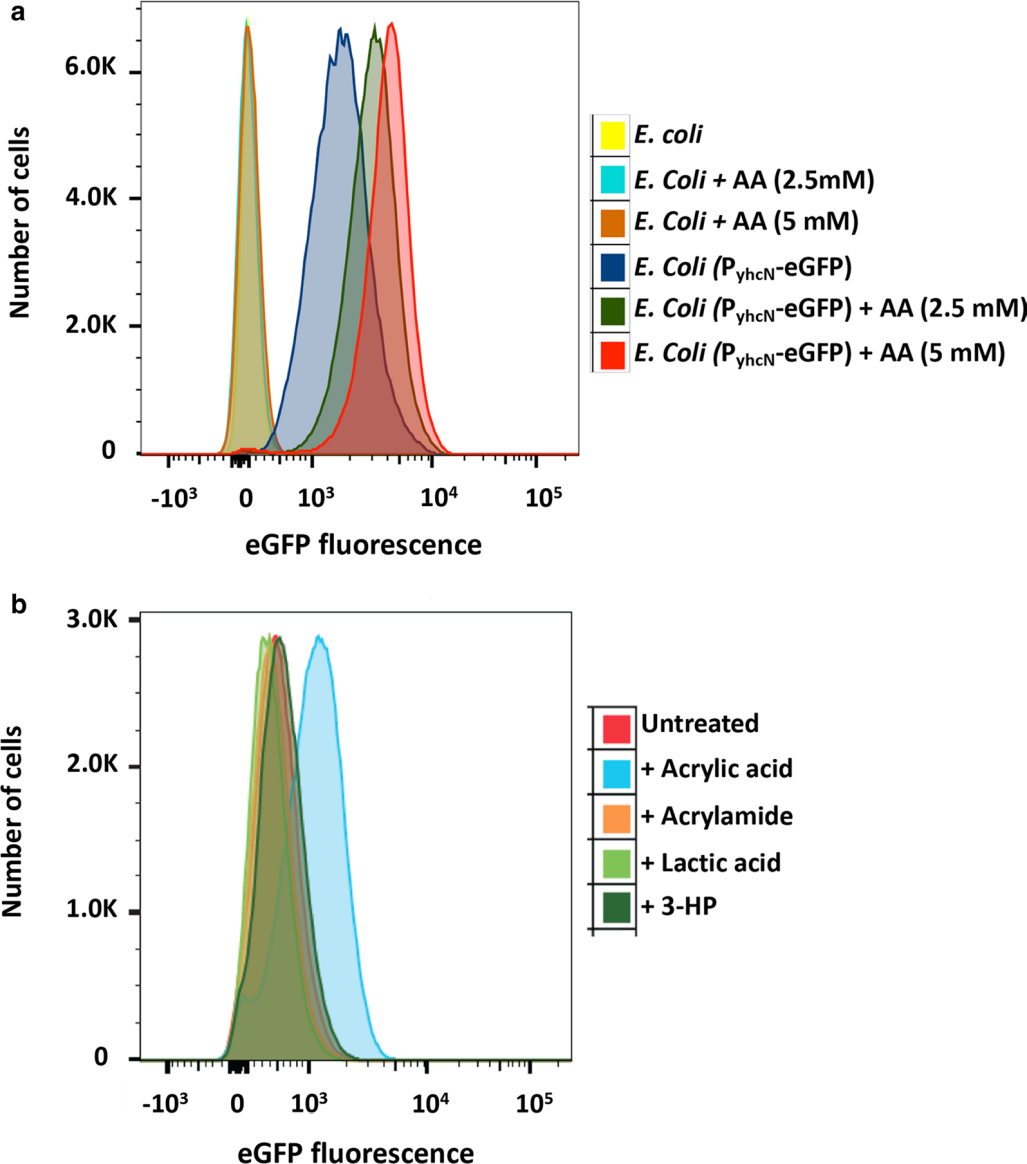
**a** Parental *E. coli* and cells expressing plasmid-encoded acrylic acid sensor were treated with indicated acrylic acid concentrations (3 h) and eGFP fluorescence measured by FACS. Traces for both control and treated parental *E. coli* cells (yellow, cyan and brown) overlap. **b** Response of P_yHCN_-eGFP sensor to indicated compounds (5 mM)

**Fig. 4:**
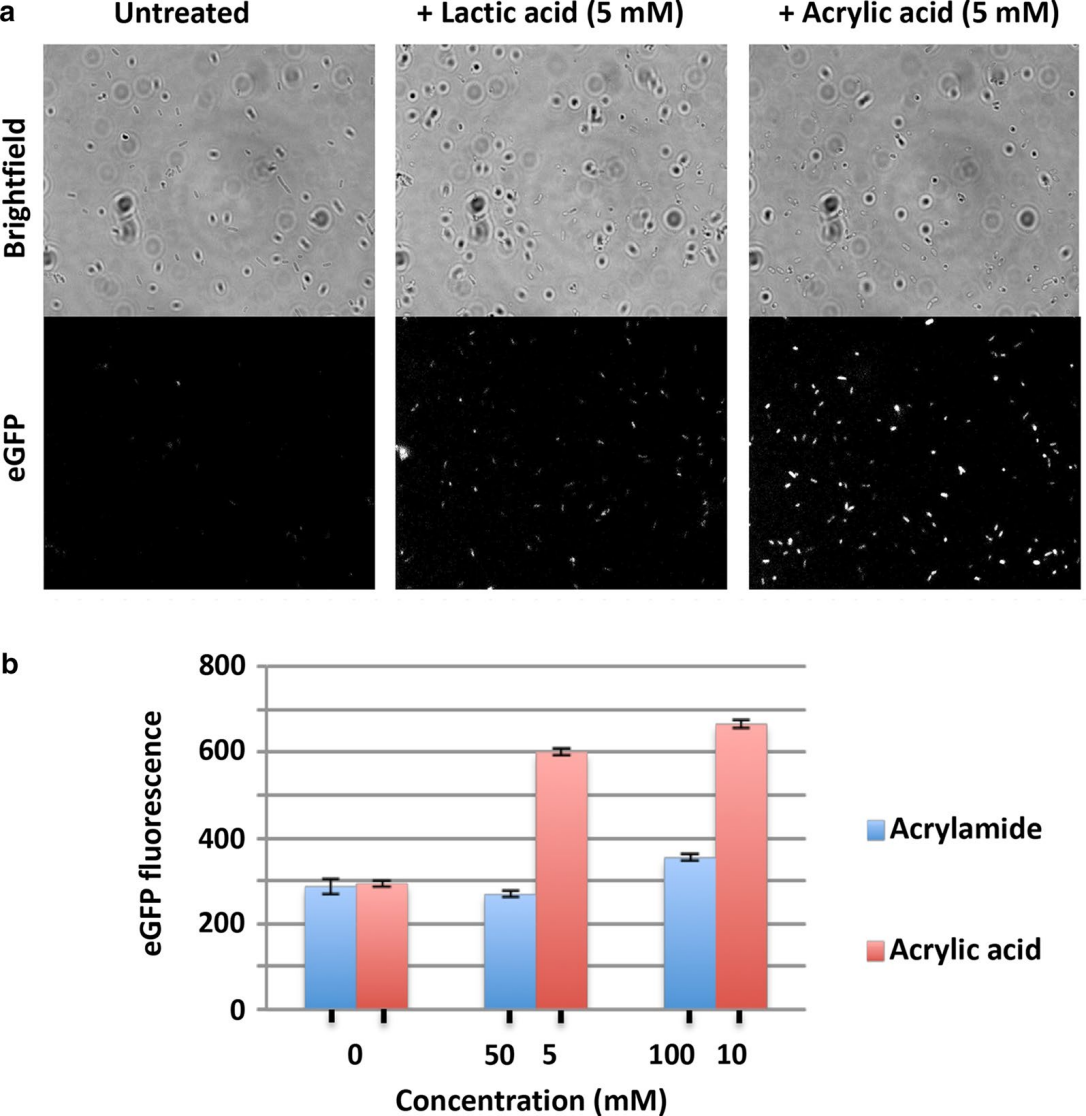
**a** Cells expressing P_yHCN_-eGFP sensor were treated as indicated for 6 h and imaged by fluorescence microscopy. **b** eGFP fluorescence of cells treated with AA (0, 5, 10 mM) or acrylamide (0, 50, 100 mM)

The P_yhcN_-eGFP sensor strain was next evaluated by FACS analysis. Cells treated with acrylic acid (0, 2.5, 5 mM) showed a dose responsive increase in eGFP fluorescence indicated by right shifting of the histogram (Fig. 3a). Treatment with the potential acrylic acid precursors acrylamide, lactic acid and 3-hydroxy propionic acid (5 mM) did not result in appreciable signal gain, confirming specificity of the P_yhcN_-eGFP sensor (Fig. 3b). This was further confirmed by fluorescence imaging of the P_yhcN_-eGFP sensor strain 6 h post treatment with AA (5 mM) which showed clear enhancement of signal over no-treatment and lactic acid (5 mM) controls (Fig. 4a). Additionally, high concentrations of acrylamide (100 mM) did not activate the sensor (Fig. 4b).

### Stable integration of the transcriptional acrylic acid sensor into *E. coli* cells

**Fig. 5:**
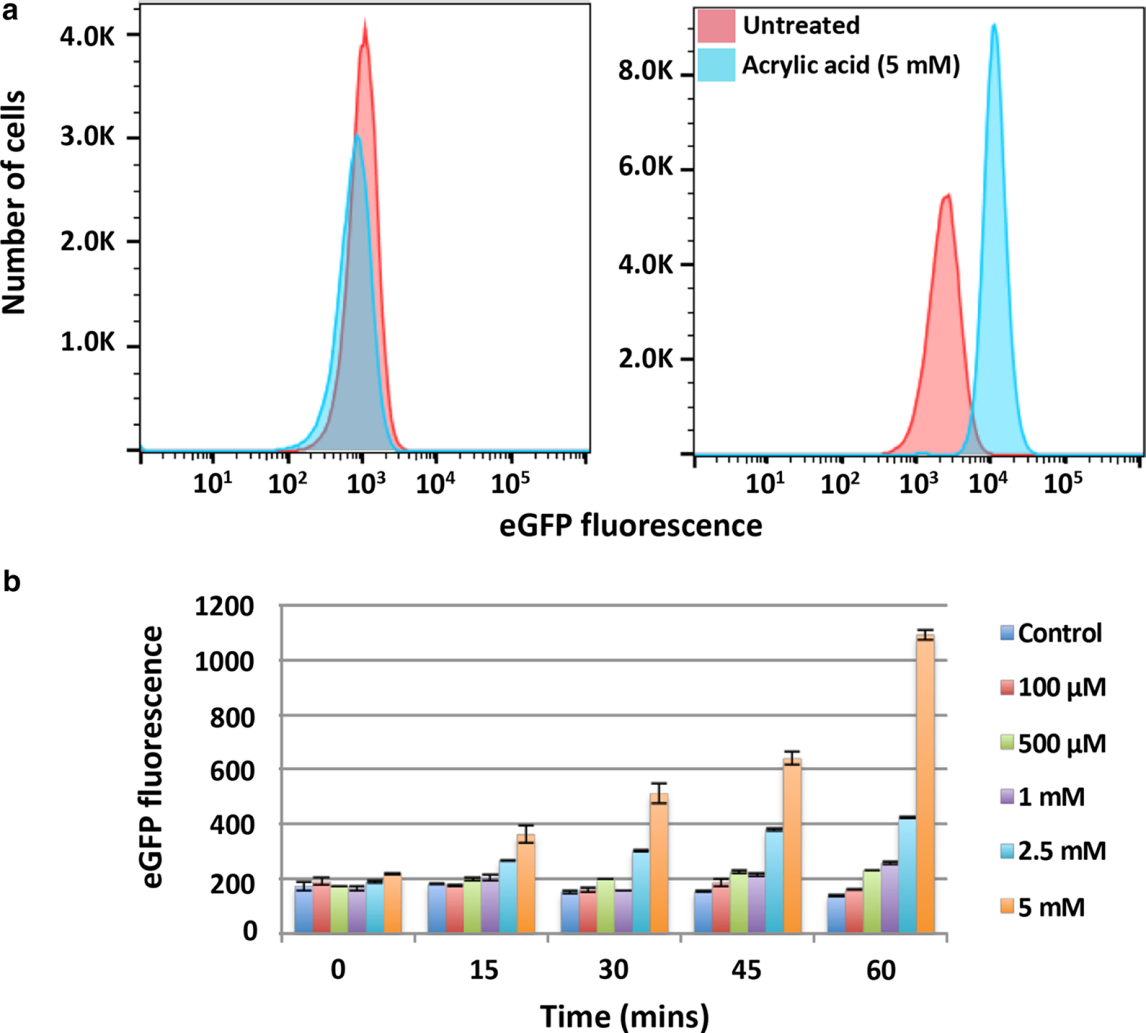
**a** Control *E. coli* (left) or stably transduced *E. coli* (P_yHCN_-eGFP)(right) were treated with acrylic acid (5 mM) overnight and analysed by FACS. **b** Stably transduced AA reporter cells treated with indicated concentrations of AA and fluorescence measured over time

The P_yhcN_-eGFP reporter cassette was next stably integrated into *E. coli* using bacteriophage lambda mediated recombination [17]. The resultant strain showed improved sensor turn-on in the presence of AA when measured by FACS (Fig. 5a). Dose-dependent signal read-out was also readily detected by fluorescence measurements using a plate reader, with relatively low concentrations (500 μM) of AA measurable after 45 min using the whole-cell P_yhcN_-eGFP biosensor (Fig. 5b). Imaging of the stably transformed strain exposed to AA showed clear analyte-dependent eGFP fluorescence (Additional file 2: Fig. S5), as observed with the plasmid-based sensor (Fig. 4a).

### Selection of improved *G. pallidus* RAPc8 amidase variants

**Fig. 6:**
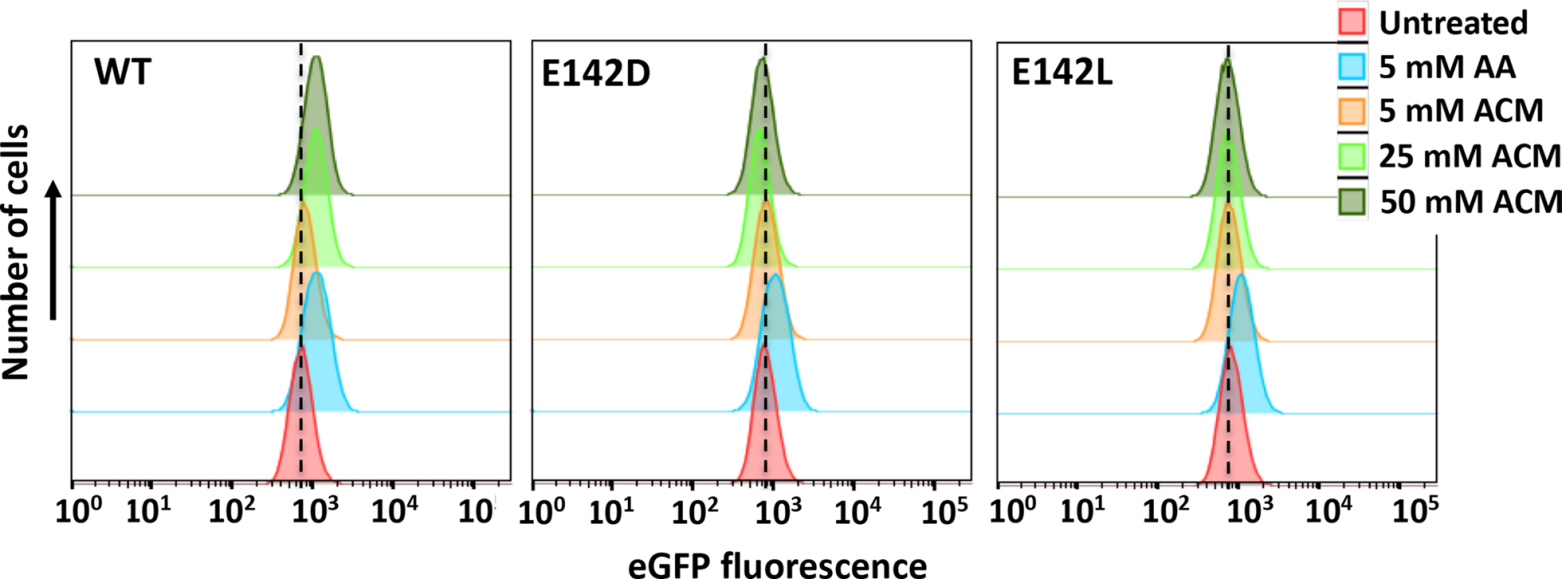
FACS analysis indicating eGFP fluorescence of stably transduced *E. coli* (P_yHCN_-eGFP) cells expressing RAPc8 amidase (WT) or the inactive E142D and E142L mutants. Cells were treated with indicated concentrations of acrylic acid (AA, positive control) or acrylamide (ACM) for 2 h prior to analysis

Acrylic acid can be made via enzymatic deamination of acrylamide as demonstrated using aliphatic amidases [18, 19]. We coupled this relatively simple pathway to AA sensor output by expressing the *G. pallidus* RAPc8 amidase [19] in *E. coli* and measuring sensor fluorescence by FACS after incubation with acrylamide. A clear upwards shift in the population of eGFP-expressing cells is observed compared to the no-substrate control (Fig. 6). This shift is not observed when the catalytically inactive E142D and E142L amidase mutants [20] were expressed. We next created a library of randomly mutated RAPc8 amidase variants (n = ~ 5 × 10^5^) and screened for improved in vivo acrylamide deamination by FACS. After 2 rounds of selection, secondary screening was carried out on 62 individually sorted clones using plate-based measurement of reporter fluorescence. The top 5 clones from this analysis were further analysed by FACS, and this confirmed 4 of these clones to show improved activity over WT (Additional file 2: Fig. S6).

**Fig. 7:**
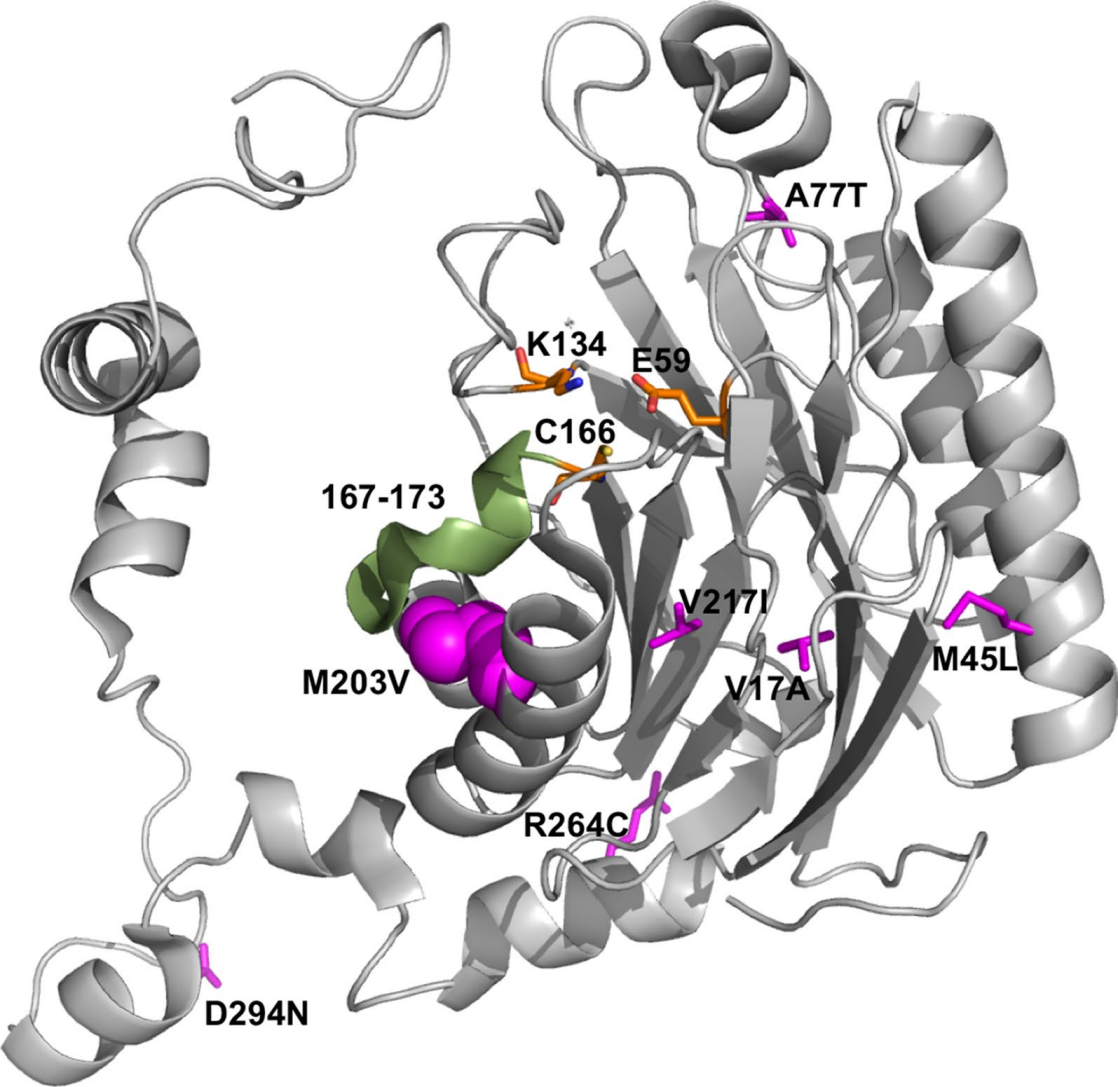
Position of mutations present in selected RAPc8 C60 and C65 variants (magenta). Residues depicted in orange (E59, K134, C166) comprise the catalytic triad. M203 is shown in spheres to highlight packing against the distorted helix (residues 167–173 denoted in green) that likely undergoes structural changes to adapt to the loss of interactions when M203 is mutated to V with a smaller sidechain (adapted from the crystal structure 2PLQ [28] and drawn using PyMOL [29])

Sequence analysis of the amidase gene in 3 of these selectants (C26, C60, C65) highlighted 5 mutations in C60 (M45L, A77T, M203V, D294N, K342E), and 3 mutations in C65 (V17A, V217I, R264C). Interestingly, whilst C26 encoded WT enzyme, two silent mutations were present in codons 208 (GCG to GCA) and 326 (ACT to ACC) that likely contributed to increased amidase expression and acrylamide turnover via codon-optimisation effects [21, 22]. Mutations in C60 and C65 were distributed throughout the enzyme’s tertiary structure (Fig. 7). Comparative sequence analysis indicated common phylogenetic variation at positions 77 (alternatively T or S) and 203 (alternatively V or I), highlighting natural selection for the A77T and M203V mutations in C60 identified here by laboratory evolution. Notably, positions 77 and 203 were correspondingly S and V in the *Hydrogenovibrio kuenenii* aliphatic amidase (84% sequence identity to RAPc8 amidase). Kinetic parameters of purified RAPc8-C60 for the acyl transfer reaction indicated ~ 1.6-fold improvement in kcat/Km over wild-type enzyme (Table 2) that was driven by an elevated kcat. The M203V mutation contributed towards this improved activity of C60, with RAPc8-M203V showing ~ 1.3-fold kcat/Km increase over WT. This is likely due to repositioning of an adjacent distorted helix (residues 167–173) that precedes C166 of the catalytic triad (Fig. 7). Whilst RAPc8-A77T displayed ~ twofold reduced Km compared to WT, this was accompanied by reduced kcat. Kinetic paramters of the RAPc8-A77T/M203V double mutant were essentially the same as RAPc8-A77T. Other mutations present in C60 therefore likely contributed to the overall improved phenotype.

**Table 2:** Kinetic parameters for RAPc8 and indicated mutants

RAPc8 enzyme variant	Vmax (uM min^−1^)	Km (mM)	kcat (min^−1^)	kcat/Km (mM^−1^ min^−1^)
WT	106.5 ± 6.1	7.3 ± 0.1	7096.3 ± 404.7	978.9 ± 63.3
A77T	66.7 ± 2.1	4.5 ± 0.2	4447.7 ± 139.2	996 ± 21.4
M203V	146.8 ± 16.7	7.2 ± 1.1	9784.7 ± 1113.3	1374.5 ± 70
A77T + M203V	64.7 ± 4.6	4.6 ± 0.4	4315 ± 308.4	935.7 ± 35.9
C60	176.3 ± 16.1	7.4 ± 1.3	11,754.3 ± 1073.3	1607.1 ± 143.5

Values represent average ± SD (n = 3)

In the selection protocol we describe there is the possibility that diffusion of AA from within cells expressing active amidase genes would co-activate the sensor in other cells expressing inactive mutants, resulting in increased false postives and higher background. Future selections could mitigate this issue by segregating cells utilising compartmentalisation [23] or performing colony-based selection on agar plates [24].

### Up-regulation of *yhcN* in response to stress

The AA-responsive yhcN gene identified in this study encodes an 87 amino acid polypeptide with a predicted periplasmic location. It has previously been ascribed to a network of *E. coli* stress-induced proteins, with its expression up-regulated by hydrogen peroxide and cadmium exposure. It is also implicated in regulating biofilm formation in response to stress [25]. Furthermore, in addition to 4 other markedly AA-responsive genes identified in this study (*yhjX, nrdH, gcd and gadA*)(Table 1), *yhcN* is up-regulated in low-pH conditions in the *E. coli* strain K-12 W3110 [26, 27]. In our experiments, the P_yhcN_-eGFP reporter was selectively responsive to acrylic acid, showing no activation by lactic acid or 3-HP. Given the similar pKa values of these acids, the differential response may arise from the different *yhcN* promoter contexts (endogenous versus synthetic construct) and/or differences in effective intracellular concentrations of these acids. As lactic acid and 3-HP are both direct precursors of AA in synthetic pathways grafted into *E. coli [*7*–*9*]*, the developed AA sensor should expedite efforts to increase yields. This could be achieved through rational pathway engineering and/or mutagenesis of key enzymes linked to selection, as shown in this study by identification of improved amidase variants.

## Conclusions

In summary, we have developed and applied a transcriptional biosensor facilitating detection of acrylic acid in vivo. This was achieved through transcriptome analysis of *E. coli* exposed to acrylic acid and validation of candidate promoters upstream of highly up-regulated genes. We further applied the biosensor to report on *E. coli* acrylic acid production linked to the simple enzymatic deamination of exogenous acrylamide. This tool should expedite both development and optimisation of *E. coli* strains capable of producing sustainable and economically viable bio-acrylic acid.

## Materials and methods

### Chemicals and reagents

All chemicals including acrylic acid, acrylamide, lactic acid, 3-hydroxy propionic acid and DMSO used in this study were purchased from Sigma-Aldrich (USA).

### Oligonucleotide primers

**Table:** 

*Real-time PCR primers*	
yhjX-F	5′-TGCTGACGCTCTCTAACTGC-3′
yhjX-R	5′-GCAATCGCTCCCCAAATCAC-3′
bhsA-F	5′-TGTCATTTGCCAGCTTTGCG-3′
bhsA-R	5′-TACGGAAAGATTTTGCGCCC-3′
gcd-F	5′-TGGTCGCAATCAGGAAGGTC-3′
gcd-R	5′-ATCGGCGTCACTTCATTGGT-3′
ybgS-F	5′-ATGTCGCGCCAAATAACGTC-3′
ybgS-R	5′-TATCCGGACAGCGACCATCT-3′
yhcN-F	5′-TCTCTTTCGGTGCATTCGCT-3′
yhcN-R	5′-TAATCTGGTAGGCCGTTGCG-3′
nrdH-F	5′-GTAACGATTGCGTTCAGTGCC-3′
nrdH-R	5′-CAGACCAGCTAAGATCGCCA-3′
prpC-F	5′-CGAGTTTAACGCCTCCACCT-3′
prpC-R	5′-TCGTAGCGTTGCTGGATCTC-3′
sufA-F	5′-CTTAGGCGTGAAGCAAACGG-3′
sufA-R	5′-TCGACTTCCGTGCCATCAAT-3′
prpB-F	5′-GCTGCCCGATCTCGGTATTT-3′
prpB-R	5′-CGCACCGGCTTTAATCATCG-3′
gadA-F	5′-CTGCTGGCATAAATTCGCCC-3′
gadA-R	5′-GTGTAGGTCACGCCGAAAGT-3′
adk-F	5′-ATCCGCCGAAAGTAGAAGGC-3′
adk-R	5′-TTACCCGCTTCCGCTTCTTT-3′
gyrB-F	5′-TGGTTGTGGTATCGGTCGTG-3′
gyrB-R	5′-GCTGAGCGATGTAGACGTGA-3′
*Primers to clone promoters and GFP into pUC19 vector*	
GFP BamHI	cgactctagaggatccATGGTGAGCAAGGGCGAGG
GFP NdeI	tgagagtgcaccatatgTTATCTAGACTTGTACAGCTCGTCCATGCCG
P_yhjX_ for	ccttttgctcacatgtCGTAACAGTCACAATTGAAACCATTAAATAAC
P_yhjX_ rev	GCCCTTGCTCACCATGGCAGTATTCCTGCAGTAATAAAAAGG
P_bhsA_ for	ccttttgctcacatgtGATGCCGTTGTACCTGGTGAC
P_bhsA_ rev	GCCCTTGCTCACCATAATAGTGGCCTTATGCAGATGAATGAC
P_yhcN_ for	ccttttgctcacatgtTCTCTGCCCCGTCGTTTC
P_yhcN_ rev	GCCCTTGCTCACCATGATTTTTACCTCGACATAATCTTTTAGCTGG
P_prpB_ for	ccttttgctcacatgtAGCGCACCGCAAAGTTAAGAAAC
P_prpB_ rev	GCCCTTGCTCACCATAGCCCATCCTTTGTTATCAACTTGTTATTTG
*Sequencing primers*	
eGFP mid rev	5′-AGGGTCAGCTTGCCGTAGG-3′
pET Upstream	5′-ATGCGTCCGGCGTAGA-3′
Duet Down1	5′-GATTATGCGGCCGTGTACAA-3′
Duet Up2	5′-TTGTACACGGCCGCATAATC-3′
T7 terminator	5′-GCTAGTTATTGCTCAGCGG-3′
ACYCDuetUP1	5′-GGATCTCGACGCTCTCCCT-3′
pETF2	5′-CATCGGTGATGTCGGCGAT-3′
petR	5′-CGGATATAGTTCCTCCTTTCAGCA-3′
*Stable integration primers*	
attP-F	cacagaattcCGTCTGTTACAGGTCACTAATACCATCT
attPSOE-R	ACATTTCCCCGAAAAGTGCCACCTGAACATCACCGG GAAATCAAATAATGAT
pSR158-F	5′-GATCCGGCTGCTAACAAAGCC-3′
pSR158-R	5′-GATTTTTACCTCGACATAATCTTTTAGCTGGG-3′
EcoliAttB-F	CTG AAA ATG TGT TCA CAG GTT GCT
EcoliattB-R	GCA ATG CCA TCT GGT ATC ACT
TEM1prom-F	TTC AGG TGG CAC TTT TCG GGG AAA TGT
TEM1prom-R	TGT GGA ATT CCT ACA CTA GAA GGA CAG TAT TTG GTA TCT GC
*Cloning RApc8 amidase WT and mutants*	
GpAmidase-F	gtataagaaggagatataCATATGCGTCATGGAGATATTAGCTCCTC
GpAmidase-R	cagcggtttctttaccagaCTCGAGTTAGTGGTGGTGGTGG
E142D-F	5′-CTTGGTGCCCCATCGAtGGGTGGTACCCTGGCG-3′
E142D-R	5′-CGCCAGGGTACCACCCaTCGATGGGGCACCAAG-3′
E142L-F	5′-CCTTGGTGCCCCATCctgGGGTGGTACCCTGGC-3′
E142L-R	5′-GCCAGGGTACCACCCcagGATGGGGCACCAAGG-3′
A77T-F	5′-GAAATGTTCGCGACAGCCaCCAGCATTCCAGGGG-3′
A77T-R	5′-CCCCTGGAATGCTGGtGGCTGTCGCGAACATTTC-3′
M203V-F	5′-GAACAGCAAATAATGgTGGCTAAAGCAATGG-3′
M203V-R	5′-CCATTGCTTTAGCCAcCATTATTTGCTGTTC-3′

### Identification of acrylic acid up-regulated genes by RNAseq analysis

#### Acrylic acid treatment and RNA isolation

*Escherichia coli* BL21 cells cultured in LB medium (37 °C) were exposed to increasing concentration of acrylic acid at the mid-log phase (OD_600_ = 0.5), and the sublethal dose determined from the growth curves. For RNAseq experiments, *E. coli* BL21 culture was treated with either 0 or 5 mM acrylic acid at the mid-log phase and the cells were harvested at 0, 30, 60 and 90 min time points. Total RNA was extracted using RNeasy Mini kit (Qiagen) following the manufacturer’s protocol, and rRNA was removed using ribo-zero rRNA removal kit (Epicentre). RNA was quantified and quality confirmed by Bioanalyzer quality analysis (RIN values > 9.0). Experiments were carried out in triplicate. Illumina sequencing libraries were generated from the RNA using the NEBNext RNA-seq library preparation kit following manufacturer’s guidelines (New England Biolabs). Each library was barcoded during PCR, libraries were then quantified by qPCR and equimolar aliquots of each library were pooled together. Sequencing was done on an Illumina HiSeq 2500 instrument.

#### Gene-based expression matrix

Cuffnorm v2.2.0, a program that is part of Cufflinks [30], was used to generate a gene-based expression matrix from the BAM files. Cuffnorm was run with the options of “–library-type fr-unstranded” and “–library-norm-method classic-fpkm”. The resulting expression matrix was normalized for library size and values were represented as FPKM (fragments per kilobase of exon per million fragments mapped).

#### Hierarchical clustering

Hierarchical clustering was performed on the FPKM expression matrix using R v3.1.0. The expression matrix was first transformed to the log-2 space before computing the distance matrix based on the Euclidean distance measure using the dist function of R. The Spearman’s rank correlation was next calculated using the cor function before being plotted using the heatmap.2 function from the gplots package of CRAN.

#### Differential expression analysis

Cuffdiff v2.1.1 (part of Cufflinks program) was used to identify differentially expressed genes at each time point. Default parameters were used except for the option of “–multi-read-correct” and “–max-bundle-frags 100000000”. A threshold of FDR < 0.05 and absolute log2 fold change > 2.0 was employed for significance. SAM (Significance Analysis of Microarrays) [31] was used to identify genes differentially expressed across time points. Analysis was performed in R v3.1.0 using the samr package from CRAN with the following options: resp.type = “Two class unpaired timecourse”, nperms = 100 and time.summary.type = “slope”. Genes having a log-2 fold change > 2.0 were assigned as up-regulated, while those having a log-2 fold change smaller than − 2 were assigned as down-regulated.

#### Analysis of endogenous transcript levels by qPCR

qPCR was carried out using the iScript Reverse Transcription Supermix (Bio-Rad). 1 µg RNA was used for a 20 µl reaction following manufacturer’s protocol. For RT-qPCR, SsoAdvanced SYBR Green Supermix (Bio-Rad) kit was used and 10 µl reactions were set up following manufacturer’s protocol using real time PCR primers for each gene analysed.

#### Plasmid construction and live cell biosensor development

eGFP was amplified using GFP-BamHI and GFP-NdeI primers and cloned into pUC19 vector. Candidate promoter regions of the top four up-regulated genes (arbitrarily predicted to be located within the first 300 bp of the non-coding region preceding the ORF of candidate up-regulated genes) (Additional file 2: Fig. S2) were amplified by PCR using P_prom_-F and P_prom_-R primers respectively and cloned upstream of the eGFP gene in the plasmid pUC19-eGFP cut using PciI and BamHI restriction by infusion cloning method. Reporter plasmid constructs contain an ampicillin resistance gene, *E. coli* origin of replication and an acrylic acid inducible putative promoter region followed by the eGFP coding sequence. To make the truncated yhcN promoter Δ108, pUC19-P_yhcN_-eGFP was cut with PciI and re-ligated.

#### Biosensor development and validation

Live cell biosensors were developed by transforming chemically competent *E. coli* BL21 cells with the four plasmid constructs containing putative AA-responsive promoter sequences. Cells were grown in LB medium at 37 °C to mid-log phase followed by treatment with AA (or other chemicals) for various time points. The cells were collected by centrifugation, washed with phosphate-buffered saline (PBS) and resuspended in the same buffer to normalise cell concentrations to OD_600_ = 1. Expression of eGFP was measured by either FACS using the BD FACSAria cell sorter (BD Biosciences) or plate reader (PerkinElmer 2104), measuring green fluorescence (Ex/Em = 488/509 nm) of 100 µl resuspended cells (OD_600_ = 1.0). Cells were also visualised by fluorescence microscopy (AxioImager Z1 upright fluorescent microscope, Zeiss) to detect eGFP fluourescence. Treated or control cells were washed with PBS and diluted to OD_600_ = 1.0. One drop of the cell suspension was placed on a microscope slide, air dried and covered with a coverslip. The 63 × oil immersion lens was used to visualise cells that were imaged with 500 ms exposure time.

#### Generation of the *E. coli* BL21 P_yhcN_-eGFP sensor strain

pET22b-Amp-attP-P_yhcN_-eGFP was amplified using primers pETF2 and TEM1prom-R to create minicircles of attP-P_yhcN_-eGFP. The C3INT-HIS-PET22b(+) plasmid [17] was amplified with petF2 and petR and the PCR products were intramolecularly ligated to produce attP-PyhcN-eGFP and C3INT-HIS minicircles. A total of 100 ng of C3INT-HIS minicircle and 100 ng attP-PyhcN-eGFP minicircle were combined and electroporated into 25 µl electrocompetent BL21 cells. The cells were allowed to recover for 1 h at 37 °C before being plated on varying concentrations of ampicillin-LB agar plates (0.07 and 0.1 mg/ml). Incubation was carried out at 37 °C for 12–14 h to allow for expression of C3 integrase and chromosomal integration of the ampicillin-resistance cassette. Positive clones were confirmed by treating cells with 5 mM AA to turn on GFP expression. Genomic integration into the attB site was confirmed by sequencing of PCR products generated using primers EcoliAttB-F and TEM1prom-R along with EcoliattB-R and TEM1prom-R.

#### Selection of improved RAPc8 amidase variants

The RAPc8 amidase gene was randomly mutated (using the Agilent Gene Morph II random mutagenesis kit) and cloned into the plasmid pDuet-Amp-P_yhcN_-eGFP cut with NdeI/XhoI. The pDuet-Amp-RapC8 amidase + P_yhcN_-eGFP plasmid was transformed into BL21 cells to create a library (~ 5 × 10^5^ transformants). The library was grown to OD_600_ ~ 0.5, induced with 0.1 mM IPTG and acrylamide added thereafter (5 mM). After 60 min, cells were diluted in 1X PBS and analysed by FACS. The top 5% and 1% brightest cells were respectively sorted in rounds 1 and 2. The amidase gene in R1 selectants was amplified by PCR prior to digestion and ligation into cut plasmid pDuet-Amp-P_yhcN_-eGFP and transformation into BL21 cells. Individual clones post R2 (n = 62) were assayed using the plate-based fluorescence assay with a subset of these showing improved activity over WT enzyme further validated by FACS.

#### Determination of kinetic parameters

Amidase activity was determined by quantifying the production of ammonia via the modified Berthelot reaction [20]. 15 nM amidase enzyme was incubated in 100 μl volume of reaction buffer (20 mM potassium phosphate pH 7.2, 150 mM NaCl, 2 mM DTT) with acrylamide titrated up to a final concentration of 40 mM. The mixture was incubated at 37 °C for 20 min and ammonia production detected colourimetrically at 670 nm using the ammonia assay kit (Abcam). Standards were prepared using ammonium chloride. Measurements were performed in triplicate and kinetic parameters obtained using the nonlinear regression curve fitting analysis in GraphPad Prism 7 (GraphPad Software, Inc., CA).

#### Expression and purification of RAPc8 amidase variants

The RAPc8 amidase constructs were cloned with a C-terminal 6xHIS tag and transformed into *Escherichia coli* BL21(DE3) (Invitrogen) competent cells. These were grown in LB medium at 37 °C and induced at OD_600_ ~ 0.5 at 30 °C with 0.1 mM IPTG for 6 h for RAPc8, RAPc8-A77T and RAPc8-M203V. Cells expressing RAPc8-A77T/M203V and RAPC60 were induced at 25 °C with 0.5 mM IPTG and incubated overnight. Cells were then harvested by centrifugation, sonicated and clarified by centrifugation. The clarified cell lysates were applied to a His-GraviTrap column (GE Healthcare) and purified according to the manufacturer’s protocol. Protein purity as assessed by SDS-PAGE were ~ 95%, and fractions were pooled and buffer exchanged into PBS buffer with 0.5 mM DTT. All proteins were concentrated using Amicon-Ultra (3 kDa MWCO) concentrator (Millipore). Protein concentration were determined using the Bradford protein assay kit (BioRad) with bovine serum albumin as standard.

## Additional files

**Additional file 1.** RNAseq data.

**Additional file 2.** Additional figures.

## Data Availability

The data and materials used/analysed in this study are available from the corresponding author on reasonable request.
