# Supplementary material for: Development and application of a transcriptional sensor for detection of heterologous acrylic acid production in E. coli
Source: Microb Cell Fact. 2019 Aug 19;18:139. doi: 10.1186/s12934-019-1185-y (PMC6699081; doi:10.1186/s12934-019-1185-y)
Supplement: Supplementary file 2 — Additional file 2. Additional figures. [file 12934_2019_1185_MOESM2_ESM.pdf]

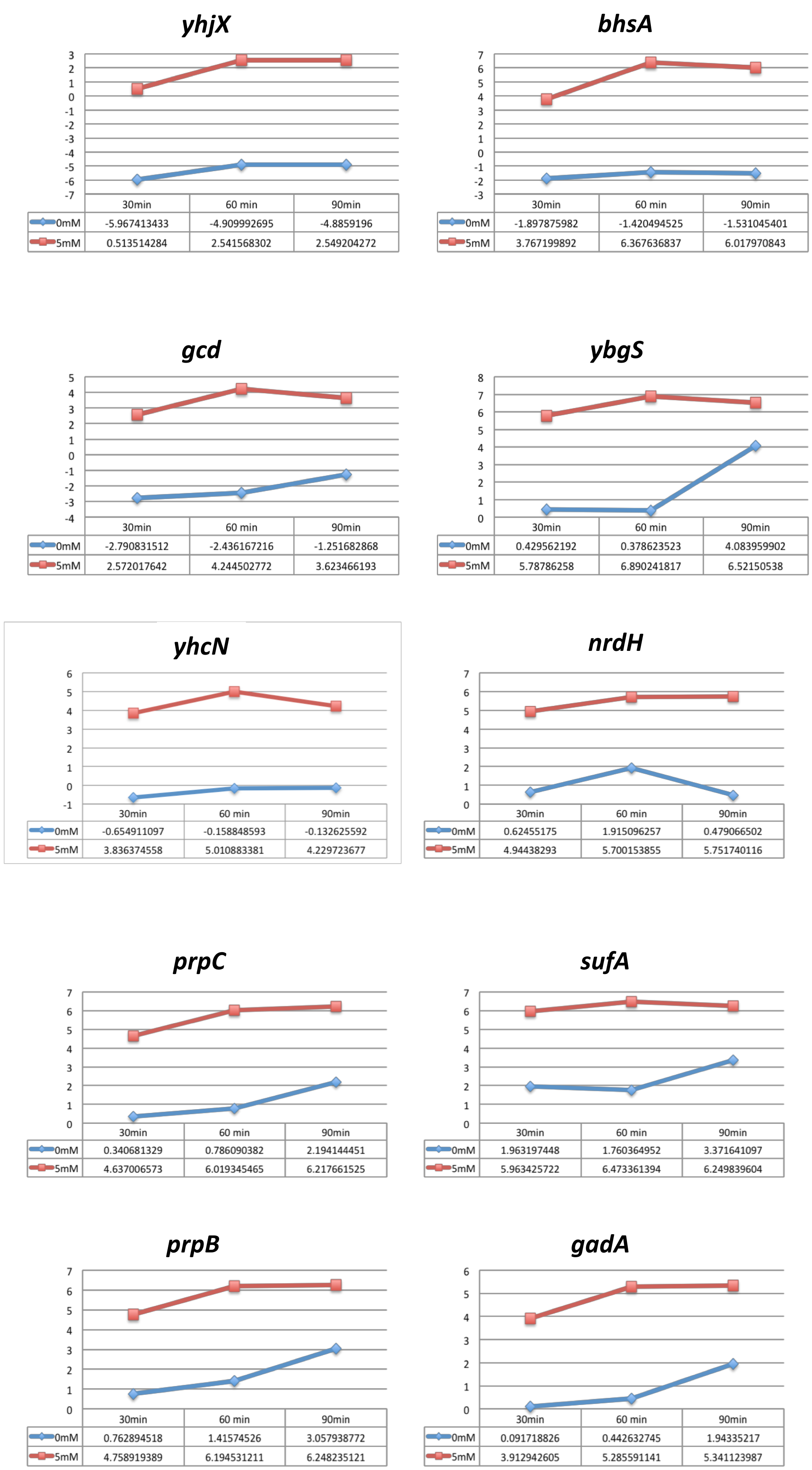

yhjX

	30min	60 min	90min
0mM	-5.967413433	-4.909992695	-4.8859196
5mM	0.513514284	2.541568302	2.549204272

bhsA

	30min	60 min	90min
0mM	-1.897875982	-1.420494525	-1.531045401
5mM	3.767199892	6.367636837	6.017970843

gcd

	30min	60 min	90min
0mM	-2.790831512	-2.436167216	-1.251682868
5mM	2.572017642	4.244502772	3.623466193

ybgS

	30min	60 min	90min
0mM	0.429562192	0.378623523	4.083959902
5mM	5.78786258	6.890241817	6.52150538

yhcN

	30min	60 min	90min
0mM	-0.654911097	-0.158848593	-0.132625592
5mM	3.836374558	5.010883381	4.229723677

nrdH

	30min	60 min	90min
0mM	0.62455175	1.915096257	0.479066502
5mM	4.94438293	5.700153855	5.751740116

prpC

	30min	60 min	90min
0mM	0.340681329	0.786090382	2.194144451
5mM	4.637006573	6.019345465	6.217661525

sufA

	30min	60 min	90min
0mM	1.963197448	1.760364952	3.371641097
5mM	5.963425722	6.473361394	6.249839604

prpB

	30min	60 min	90min
0mM	0.762894518	1.41574526	3.057938772
5mM	4.758919389	6.194531211	6.248235121

gadA

	30min	60 min	90min
0mM	0.091718826	0.442632745	1.94335217
5mM	3.912942605	5.285591141	5.341123987

Fig. S1
RNAseq data for the top 10 AA-responsive genes showing relative expression levels over indicated times in presence (red) or absence of AA (blue).

yhjX
CGTAACAGTCACAATTGAAACCATTAAATAACAATAGTTGTGGCGATAGTGGGTG
CTAACTTACCAAATAATAAATTTGGTGAATAATTGTCGCGTCATTCATTCCTGAACT
AAGGCATTTCATTCCGTTCTGATGGCATTTCATGCCGTTTTTCCCCAGGCATAAAG
TGCACTTCGTTATGGTTGTCGGCAGAGATTTTTCCTTTTTATTACTGCAGGAATACT
GCC

bhsA
GATGCCGTTGTACCTGGTGACTGTGAATGAAAGGTTATTATAAAAATAATCACCTC
CGTTCACCAGTCCAGATCCCATAAAAATAATTGCTTTCTATTTAACTGAAATTTAAA
GATTTTTAAATTAATTAATGATTGTTATAAAAAATATCTTGTATGTGATCCAGATCAC
ATCTATCATTTAGTTATCGATCGTTAAGTAATTGCTTGCGACGTCATTCATCTGCATA
AGGCCACTATT

yhcN
TCTCTGCCCCGTCGTTTCTGACGGCGGGGAAAATGTTGCTTATCCCTCTCAACCC
CCTGCTTTCCCCTGCGATTAATTTAACGAATAGTGCGTTTTACTGCGACATGTCATT
CACACAATGAATACATAAGGTAAAAAAAGCACATTATGCAAAATTCATTATCTAATT
GAAAAAACTAGAATTAACGATAAATAACCGTATTTTTAATTCTTTTTTGTTATTAAAA
TTCACATTTTTAACACTTAGTATCAACTGAAACAGTTAGCGCGGTATTAATTAGCTC
AATAATTAGTGTATACTTGATTTTGTGATATGGGTCACGAAACAAAGGCCCAGCTA
AAAGATTATGTCGAGGTAAAAATC

prpB
AGCGCACCGCAAAGTTAAGAAACCGAATATTGGGTTTAGTCTTGTTTCATAATTGT
TGCAATGAAACGCGGTGAAACATTGCCTGAAACGTTAACTGAAACGCATATTTGC
GGATTAGTTCATGACTTTATCTCTAACAAATTGAAATTAAACATTTAATTTTATTAAG
GCAATTGTGGCACACCCCTTGCTTTGTCTTTATCAACGCAAATAACAAGTTGATAA
CAAAGGATGGGCT

***yhcN* Δ108**
CATTCACACAATGAATACATAAGGTAAAAAAAGCACATTATGCAAAATTCATTATCT
AATTGAAAAAACTAGAATTAACGATAAATAACCGTATTTTTAATTCTTTTTTGTTATTA
AAATTCACATTTTTAACACTTAGTATCAACTGAAACAGTTAGCGCGGTATTAATTAG
CTCAATAATTAGTGTATACTTGATTTTGTGATATGGGTCACGAAACAAAGGCCCAG
CTAAAAGATTATGTCGAGGTAAAAATC

Fig. S2
Nucleotide sequences of promoter elements for the indicated genes used in this study.

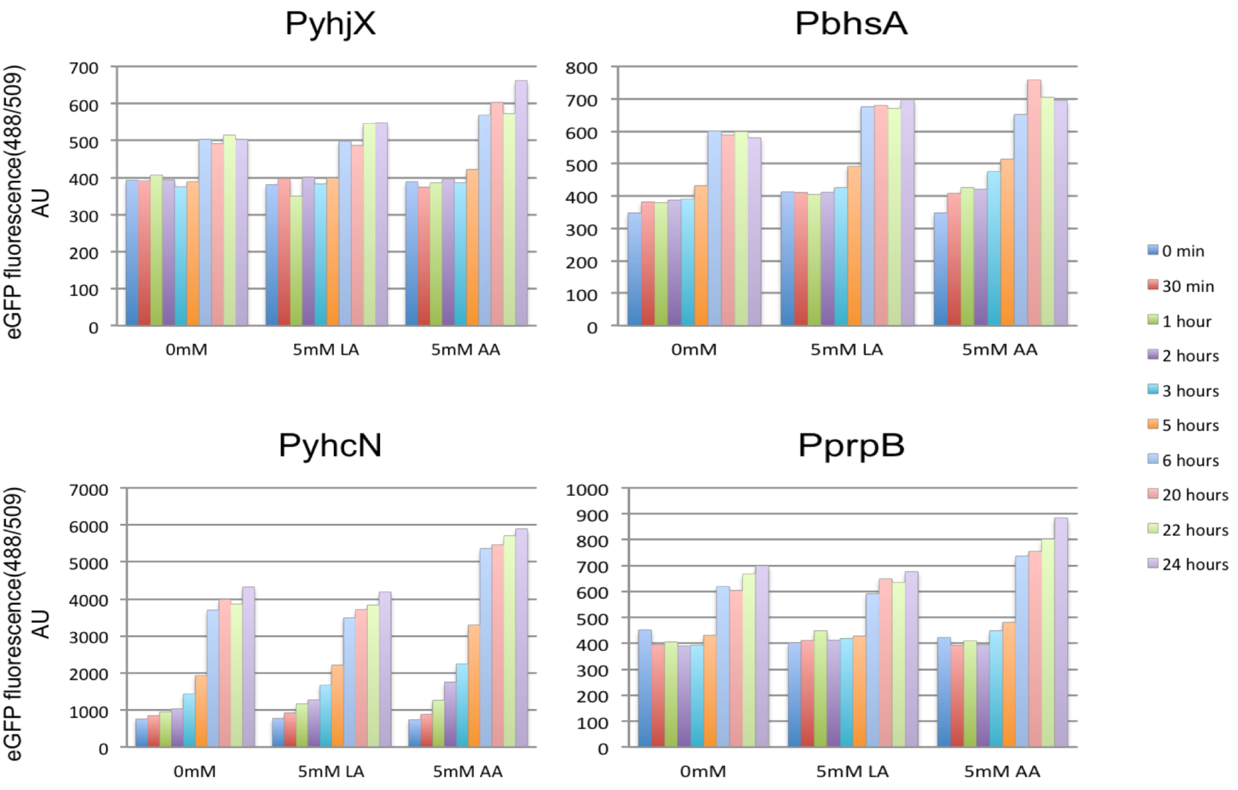

Fig. S3

Sensors (P_{yhjX}-eGFP, P_{bhsA}-eGFP, P_{yhcN}-eGFP and P_{prpB}-eGFP) expressed in *E.coli* cells treated with 5mM acrylic acid. eGFP fluorescence was observed over 24 hours.

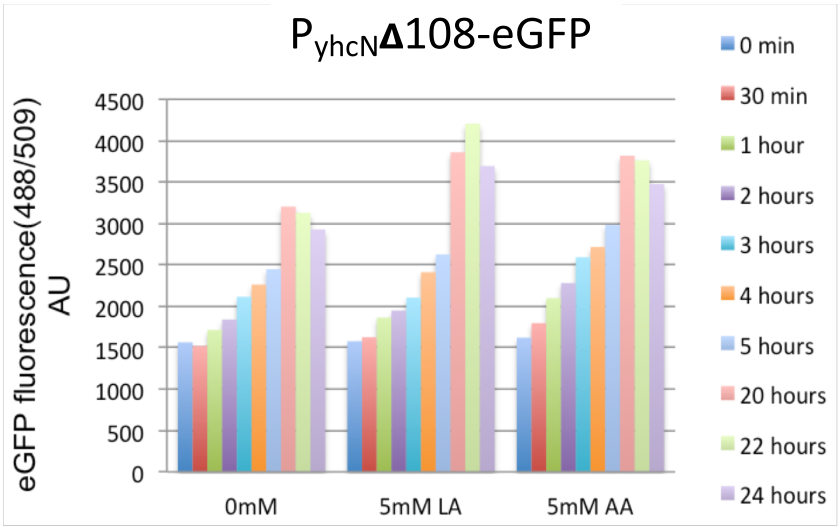

Fig. S4

The *yhcN* promoter in P_{yhcN}-eGFP was truncated by removal of 108 bases at the 5' end to generate P_{yhcN}**Δ**108-eGFP. Cells harbouring this construct were treated with 5mM acrylic acid (AA). Controls include untreated and 5mM lactic acid (LA) treated cells. eGFP fluorescence was measured over 24 hours.

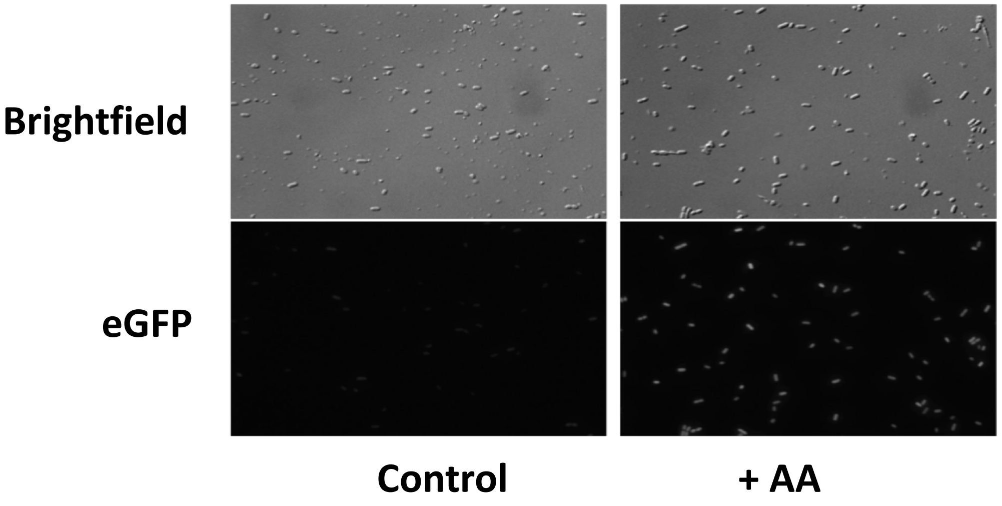

Fig. S5
E.coli cells stably integrated with the acrylic acid sensor shows eGFP fluorescence when exposed to acrylic acid (AA) (5mM) for 4 hours.

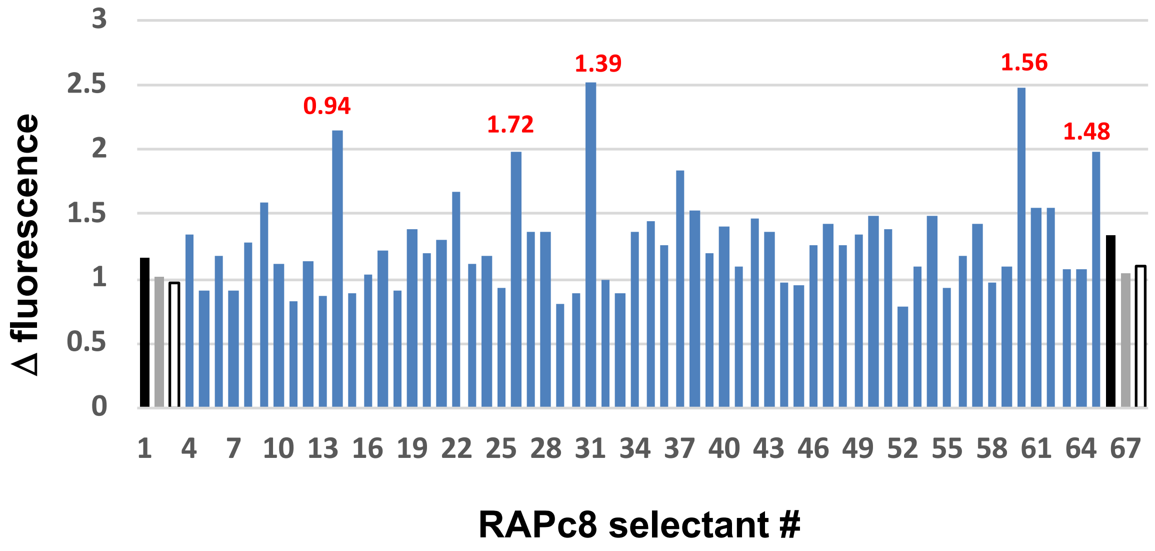

Fig. S6

Secondary assay indicating AA reporter gene activity for indicated RAPc8 selectants. Cells were treated with acrylamide (25mM) and fluorescence measured after 2 hours. Black, grey and white bars respectively indicate values for cells expressing WT RAPc8 and inactive E142D, E142L mutants. Values highlighted in red denote fold change in mean cell fluorescence over cells expressing WT enzyme determined by subsequent FACS analysis of top 5 selectants.
